# Moths on the Flatbed Scanner: The Art of Joseph Scheer

**DOI:** 10.3390/insects2040564

**Published:** 2011-12-14

**Authors:** Stephen L. Buchmann

**Affiliations:** 1 Departments of Entomology, Ecology and Evolutionary Biology, The University of Arizona, Tucson, AZ 85743, USA; E-Mail: buchmann.stephen@gmail.com; Tel.: +1-520-797-2638; 2 The Pollinator Partnership, 423 Washington St., San Francisco, CA 94111-2339, USA

**Keywords:** moths, Lepidoptera, flatbed scanners, scanography, entomology, art, Joseph Scheer, Institute of Electronic Arts, Alfred University

## Abstract

During the past decade a few artists and even fewer entomologists discovered flatbed scanning technology, using extreme resolution graphical arts scanners for acquiring high magnification digital images of plants, animals and inanimate objects. They are not just for trip receipts anymore. The special attributes of certain scanners, to image thick objects is discussed along with the technical features of the scanners including magnification, color depth and shadow detail. The work of pioneering scanner artist, Joseph Scheer from New York's Alfred University is highlighted. Representative flatbed-scanned images of moths are illustrated along with techniques to produce them. Collecting and preparing moths, and other objects, for scanning are described. Highlights of the Fulbright sabbatical year of professor Scheer in Arizona and Sonora, Mexico are presented, along with comments on moths in science, folklore, art and pop culture. The use of flatbed scanners is offered as a relatively new method for visualizing small objects while acquiring large files for creating archival inkjet prints for display and sale.

## 1. Introduction

Flatbed scanners are routinely used around the world millions of times daily, found in virtually every business office and many homes. They are routinely used to make copies of flat objects, typically paper documents including scanned receipts for accounting or similar purposes, usually as black and white or grayscale images. Once in a while, someone places a color photograph on the scanner glass, closes the lid and makes a color scan for importing into Photoshop™, LightRoom™or another image-editing program. Or, someone attempts to copy a color photo on a page torn from a magazine and is disappointed with the results due to the halftone printer's dots because their scanning software does not have a de-screening algorithm. Boring! This barely scratches the surface of what modern flat bed scanners, and their software, can produce for working artists, scientists, naturalists and students. Scanned objects need not be monochromatic or paper thin. A few scanner models have enhanced depth-of-field capabilities and can accommodate thicker objects, up to several centimeters. Their unique sunlight-balanced even illumination, as the fluorescent tubes pass below scanned objects, yields dramatic lighting difficult or impossible to reproduce with copy stands, studio lights, cameras and lenses. When viewing a print made from a scanned image, a first-time observer often thinks the image was created photographically, but something is not quite right. Lighting is everything, and scanner light is uniquely different from using film or DSLR cameras.

Think of flatbed scanners as wide-field microscopes capable of higher magnifications than macrophotography, and easier to use than setting up tripods, cameras with close-focusing lenses and light stands in a studio. This article is about using flatbed scanners for natural history imaging, and the inspiring work of one man, Alfred University artist Joseph Scheer, a fine arts print-maker who turned his attention to the dark side, the nocturnal world of moths from Arizona and Sonora, Mexico [1-3]. Representative images, both scanned and photographic, of Scheer's moths from his Fulbright year (2010/2011) in Sonora, Mexico are illustrated along with discussions of the interface and interactions between the science of entomology and the visual art of scanning and printmaking, and his interactions with scientists, other artists and writers. This provides another example of the imaginative use of insects, as iconic subjects in pop culture, art, and music for this special issue of the journal Insects.

## 2. Flatbed Scanning Technology

Although almost any flatbed scanner will work for capturing images of natural history specimens or original art, those that are large, thick and heavy work the best. They are stable and do not vibrate during the long acquisition times needed at the highest resolutions. Scanners that are one or two centimeters thick have a different internal optical mechanism that makes them unsuitable for natural history scanning (NHS). When shopping for a scanner to purchase, look for established manufacturers producing high-end graphics scanners. This type of pre-press graphics art scanner was originally used to capture/scan fine art originals, which were then printed on the pages of fine art large format coffee table books. Scanner manufacturers including Agfa, Hewlett Packard, Epson and Creo/Kodak come to mind. Some of my favorite workhorse scanners are listed in Table 1. The technical specifications that set these scanners apart from normal document “copy” scanners include professional features such as:

- Thickness. The ability to image thick specimens placed on the scanner glass. This might include objects from 1 to 5, 6 or more centimeters tall. Depth-of-field increases when scanning at lower dpi settings, e.g., 300 to 600 dpi. Some scanners (e.g., Epson, Creo/Kodak) can focus at user-defined heights (to a precision of 0.1 mm) and these depth slices can later be assembled manually in Photoshop or with hyper-focal software to create images all or mostly in focus through the entire specimen. This is a software cheat that breaks a well-known law of optics in microscopy. That is, depth-of-focus decreases as magnification increases.
- Extreme resolution. Many graphic arts scanners will operate at higher resolutions than inexpensive home scanners. Typically, you'd want a scanner that can produce true 2400 × 4800 dpi optical resolution. Many of the high figure dpi estimates on company websites or sales brochures are hype: their scanners cannot handle those optical extremes or if they do, will produce unsharp images. Beware of inflated claims. Higher resolutions, up to 14,000 dpi through software interpolation usually result in inferior images compared to excellent true optical resolution. On the other hand, the very best scanners, including the Scitex/Creo brand developed in Israel, and now owned by Kodak Inc., Rochester, NY, USA, achieve true optical resolutions of 5,600 dpi across the full scanner glass. There are no “sweet spots” of maximum resolution. Some of these scanners have electronically cooled CCD detector arrays to achieve their high optical resolutions with extremely low digital noise. Fine craftsmanship and high technology come at a price, and some models cost as much as a luxury car. There are less expensive professional art scanner solutions from other companies, including Epson. To put these super scanners into perspective, screen resolution on computer monitors is typically 72–100 dpi, and fine art prints are usually images at 240–300 dpi printed on inkjet printers at 1,440 or 2,880 dpi resolutions. Extreme scanners will produce large file sizes (300 Megabytes to 1 Gigabyte per image) that can be printed as very large fine art prints with stunning, near microscopic detail. Magnifications of 60× life size are achievable.
- Color and Bit Depth. High-end graphical arts scanners are optimized to acquire image information with smooth gradations up to 48-bit color. Human vision cannot detect such subtle differences in images that may include scanned captures of over 4 trillion colors and 4,096 gray levels.
- Shadow Detail. One of the best features of the graphic arts scanners is their ability to record shadow detail, resolving details in the darkest part of an object. The technical measure of shadow detail is called D_max_ and the best flatbed scanners achieve 3.8 to 4.0 D_max_ values. These high D_max_ values allow for accurate and precise color matching of scanned objects even in the shadowed areas.
- Beautiful Light. The Xenon lamps in flatbed scanners and that objects are scanned slowly from below, combine to create a unique lighting not duplicated using photographic techniques. Depth-of-field can be enhanced by scanning at various heights and combining images later. Scanners are operated with the scanner lid up or removed and in a darkened room and often with colored backgrounds or with boxes covering the specimen and shielding it from extraneous light.

## 3. Scanning Natural History Objects

Flatbed scanners are not camcorders. Living specimens cannot be scanned or strange rainbow-like streaks will appear in the resulting image. Specimens should be freshly killed or dried. Very light-weight specimens can shift due to air currents or vibrations produced during scanning. For these reasons, it is often necessary to tape or weight down specimens using glass or metal washers where they will not show on the scan. Microscope slides are useful for flattening insect wings and holding them in place.

I often use small pieces of painter's tape to affix the slides to the scanner glass since they do not leave a residue. No matter how carefully you scan, expect tiny dust particles or lint, especially when scanning objects against a black background. Unless you live and work in a silicon chip factory clean room, expect to spend minutes or hours touching up each image with the healing brush tool in Photoshop™. Clean the scanner glass between specimens using liquid eyeglass cleaner, and soft paper towels. Follow this with a final cleaning with one of the new microfiber cleaning cloths from 3M or other manufacturers.

Try to scan in a still room with no fan or AC currents, especially with light-weight specimens including insects. Dried insects and other specimens that have been stored for any length of time will themselves have surface contamination. Compressed air can be used gently to clean the surfaces, while gentle washing will be needed for others (e.g., fossils, leaves). Capturing the life colors of certain insects is especially difficult. Dragonflies often have turquoise blue colors known as Tyndall Blue. These are structural colors based upon hydrated living tissues. At the moment of death, these brilliant pigments begin to fade. The same pigmentary fading, to a dull brown, happens with the eyes of native bees, which in life can be gray, green, blue or even red. For dragonflies and bees I try to scan the specimen within 30 minutes to prevent fading. With extreme resolution scans (5,600 dpi or greater) on large specimens, the life colors may actually begin to fade during the progress of the scan. Some insects, including most beetles, have structural or pigmentary colors that are mostly stable and long-lasting even in dried museum specimens, decades old. Butterflies and moths may hold their scale colors without fading or color shifts. Exceptions are some delicate green geometrid moths which may have to be frozen (freezing is a great option in general for lepidoptera if you are not going to scan them immediately).

Plant specimens are typically easier to scan than animal subjects and can be scanned fresh or dried. Freshly picked flowers are wonderful subjects for scanography. They can be placed directly on the scanner or propped up using various means. An ingenious method used by one scanner artist (Robert Creamer of the Smithsonian Institution) is a wooden or plastic frame painted black with a roof so that floral stems tied to strings are supported in vertical positions or any angle above the scanner glass. Elaborate collages of flowers and found objects can be carefully arranged across the entire scanner glass. Flattening plants can be done by placing them between pages in a thick telephone directory for a few hours, then arranging on the scanner. If you do not already have a regular botanical plant press, it's possible to make or buy one (visit your local botany herbarium for examples). Pressed wildflowers, florist shop, or garden flowers on full sized herbarium sheets or smaller notecards are excellent subjects for scanning. Compared to fresh flowers, some of their colors fade but the flattened look of pressed flowers has a beauty and appeal all its own. For other botanicals, including sliced fruits and vegetables, a wet look results from applying a thick layer of glycerine to the samples and carefully avoiding forming small bubbles. Dams of clay or plasticine can be used to confine the liquids. Small transparent trays of Plexiglass or thin glass (1/4 inch) can be made using silicone aquarium sealant, which can hold specimens (e.g., cut flowers) for an underwater look in the resulting scans. Many unique effects are open to the imaginative scanner operator.

Heavy specimens, including minerals, slabs, crystals or fossils, can be scanned but require care in placement so that the scanner glass is not scratched or broken. Some scanner artists place a protective sheet of acetate or other film between the specimen and scanner glass. Experimentation with types of plastics are necessary to determine which ones to use, because some may produce Newton's rings, a type of interference pattern caused by reflection of light between two close surfaces (as happens often between sheets of glass), or that contain minute surface scratches.

Moths and butterflies present unusual problems for scanning unless you are scanning specimens with wings upright over the body axis and only want to digitize undersides of the wings. Lepidoptera are best spread upside down on sheets of foam for scanning. Details of how artist Joseph Scheer does this are given later.

## 4. Joseph Scheer and His Moths

I'd put flowers and a few insect specimens casually on flatbed scanners as early as the 1980s but never pursued those artistic endeavors or realized the full potential of the scanners as “wide field microscopes” with unique specimen-illuminating capabilities. It was not until I saw the May, 2002 issue of National Geographic Magazine [3] that my view of scanners changed dramatically and instantly. In a feature article entitled “Uncommon Vision” written by Lynne Warren and illustrated mostly with scanned moth images by Joseph Scheer, I saw the true power of the high end graphic arts scanners, and what they could do in the hands of the right person. Here were extravagantly colorful and exotic moths enlarged for adults, not just kids, to appreciate. Here were small moths enlarged 2,700% by a scanner extracting 67 million data points of color information from every square inch. Scanning at that resolution could take 30 minutes or more even for a small specimen and the file sizes were huge, hundreds of megabytes. From these scans, Alfred University artist (also co-founder and co-director of the Institute for Electronic Arts at Alfred) Joseph Scheer made and continues to make fine art inkjet limited edition prints on an Iris CMYK four color archival printer. The prints are brilliant in color and huge, often 34-by-46 inch (86-by-116 centimeter) wide prints on handmade French or Chinese watercolor papers. It sounds easy but every step from field collecting, spreading, scanning and working on the files takes many hours, even days of work. As Joseph says “Color correcting the scan, adjusting the printer so the final image truly matches the moth. It has to be perfect [4].”

I was mesmerized by the look of the scans and those small insects, in Josephs' case the microlepidoptera often with wingspans of only a centimeter could be imaged and printed so large while rendering perfect microscopic details of scale colors, texture and patterns. I sent a number of electronic mail messages to Scheer and got responses to queries about the type of scanner he was using and how the moths were prepared for imaging. I wanted one of those scanners. Getting my own Creo would have to wait, however, until I could afford one, even a refurbished instrument.

For the past 14 years, Joseph Scheer has traveled widely, to 7 countries in search of moths (Australia, Canada, China, Costa Rica, Mexico, Switzerland and USA) operating mercury vapor lamps and blacklights in front of cloth collecting sheets, finding tens of thousands of moths and selecting ones that were artistically intriguing, rare or scientifically interesting, to spread, scan and print. During this time he's scanned perhaps 3,000 species of moths, about 1,000 alone near his home in New York State, and made numerous inkjet prints for exhibition, demonstrations, gifts and sale in galleries. The exact number of moth species and individuals scanned is unknown; Scheer does not keep count. Maybe he does not want to know because even with these herculean labors there are so many left to collect, spread, scan and print. With about 150,000 moths formally described in the taxonomic literature, outnumbering butterflies by at least eight to one, he's not going to run out of scaly subjects for scanning anytime soon. Single-handed mostly self-funded with a few grants, he's documenting biodiversity of one of the largest insect orders, something even a huge international team with massive funding would not attempt to do.

A fine arts print maker by training and desire, Joseph had no formal training in the biological sciences and specifically not in entomology or the order Lepidoptera. Scheer has become an extremely capable and imaginative scientist, making careful observations on the natural history of moths, many new to science. He can hold his own in the jargon-rich parlance of Latin and Greek-derived scientific names for his moths. He knows where they live, what time of the year to find them, their food plants and often raises them “sleeved out” in the field, or in the laboratory to obtain perfect specimens for scanning. I'll let him tell his own formative moth story with a quote from his “Night Visions” book published by Prestel [4].

**Figure 1:**
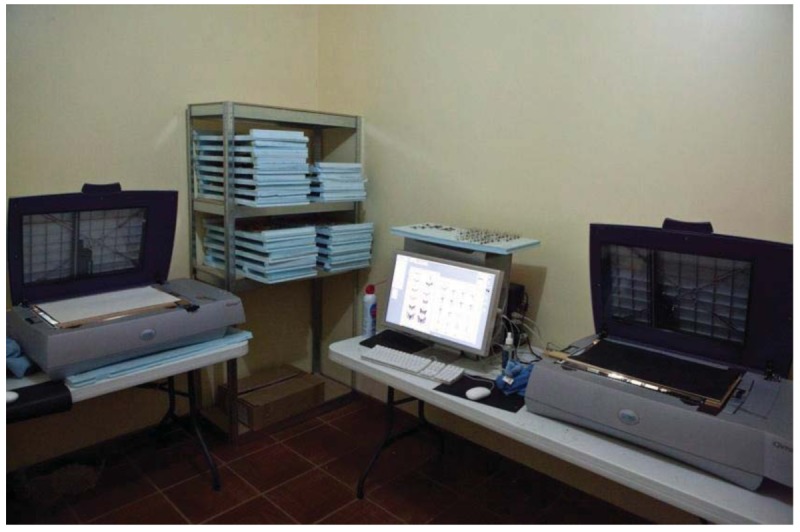
Two Creo scanners operating to process the night's catch.

“Soon after the newly formed Institute for Electronic Arts received its first high resolution scanner from Scitex (now Creo Corporation) its technician came and calibrated it and pronounced it ‘ready to scan.’ As there was a small gnat flying around a plant near the scanner, I quickly grabbed it, threw it onto the new device, and proceeded to scan it at maximum resolution. When the scan was finished and I opened the file on the computer, what I saw was incredible. This little creature had metallic pearlescent wings, hairs all over its tiny body and two beautiful, multi-faceted compound eyes. I never had looked at a gnat before with any interest, dismissing them as minor nuisances, unworthy of my attention. This experience opened a whole new world for me. I became aware of the complexity of the many insects surrounding us all the time. I began to think of the many things buzzing or crawling around my feet in the vegetation as I walked down the street [4].” The year was 1997. Eventually the Israeli company Creo was acquired by Kodak who now manufactures and markets their line of scanners (see Figure 1).

Along with the original Scitex flatbed scanner, the IEA division within the art depar™ent at Alfred University had acquired an Iris printer. These large four-color inkjet printers were originally designed as color-proofing devices for coffee table art books. They soon became highly sought-after printers for artists creating digital files who also wanted to make colorfast large prints for exhibition and sale. Many artists experimented with the early Iris printers and quickly learned that they could print on a wide variety of papers, including handmade papers and thicker stock. As a print-maker, Scheer had always sought out rare and beautiful handmade papers made in France, China and other countries. The technological pairing was established and Joseph began his immersion into Creo scanning and Iris printing. Although no longer manufactured, Joseph acquires working or non-functioning Iris printers at great savings on eBay. Originally, the Iris printers were about $250,000 each. In his classes and for his graduate students Scheer teaches the use of other printers including those made by Epson. He used Epson printers to make 8 feet tall prints of Arizona hawkmoths (the genus *Manduca* for exhibition originally in a show at the University of Arizona.

After original scanner, he began looking for exciting specimens to scan. For years he'd kept a saucer of errant bugs on his desk at school. These had flown in open windows or died early deaths in window sills. Many were dusty or had died in grotesque unnatural positions, but were worth a try. After these early tests, he quickly set a goal of 200 species and wandered campus and rural areas in NY collecting specimens. All were scanned but none really intrigued him, except the moths. He kept coming back to moths, their beauty, delicate nature, outlandish shapes and amazing colors. They called to him. As he's told me more than once “they are really much nicer than those ugly butterflies everyone likes.” That pretty much sums up his disdain for the diurnal side of the force, the butterflies which are over-researched and touted again and again compared to their species-rich kin, often maligned sisters, the moths. Soon, moths were the only quarry of any real interest and Joseph dove into moth collecting, modifying entomological techniques to suit his scanning needs, and seeking out moth texts (old standards like Holland's Moth Book [5] while seeking advice from professional entomologists at the Smithsonian Institution and other museums. Nobody, not even the professionals, knew much about moth food plants, their intricate life histories, when they flew or where they lived. But all agreed on one thing, there were an awful lot of them. Just look at any porch light on a warm summer's night, especially with a magnifying glass in hand. That's biodiversity!

## 5. Attracted to Light: Collecting in the Field

There's really only one way to get moths, lots of them, in pristine condition for scanning and making prints. You have to go into the field and collect them yourself. Forget about borrowing specimens from a museum, especially a world-class institution. Museum specimens come on stainless steel silver or black insect pins with a little plastic knob on top. Below, is a fragile bone-dry “look at it crosswise and it breaks” specimen, a unique record from a time, place and ecological setting, a habitat and season where it lived with huge numbers of its kind, or perhaps it was an errant, a loner, a singleton in a larger bountiful night's catch. Many insect museums that previously loaned out specimens to systematists for studies including generic revisions no longer do so, because fragile and rare type specimens have been lost or damaged in transit. Some museums now have digital representations of type specimens “e-types” which borrowers can look at online. Not quite the same as turning a specimen between your fingers under a microscope. So, forget asking to borrow a Schmidt box of colorful moths and taking them off their pins, even though that technique, called relaxing, is possible. Curators will not let you do it. Therefore, Joseph and a few other scanning aficionados venture out into the wilds with bed sheets, bright mercury vapor lights, collecting containers and lethal killing agents. Collecting moths, or “mothing” as Joseph Scheer fondly calls it, “lacks a definition in the dictionary but the people who collect, study and rear moths know this term well. It describes an activity that includes aspects of both fishing and husbandry but as to how they pertain to moths [4].”

Since prehistory, everyone is aware that moths sometimes fly directly toward a candle flame or the wick of an oil lamp, or a wood fire and often ignited, emolled in flames. Surprisingly, there is no universal agreement among biologists for the explanation of why moths are attracted to manmade light sources or circle flames *etc*. The most popular theory is celestial navigation or transverse orientation. By maintaining a constant angle with respect to a bright celestial object like the moon, moths can fly in straight lines and arrive at their destinations, if they have a destination. Many moths, especially males, are guided by scant sex molecules (sex pheromones) of a calling female and presumably no celestial navigation is used. If, however, a small finite light source intervenes, your porch light or a candle flame, the moth instinctively attempts to correct and makes ever sharper turns, angling eventually into the light source itself. It's also been suggested that moths may be trying to stay safe and under cover by moving to dark visual areas called Mach bands. The Mach band region corresponds with darker areas of the sky. No matter, they end up in dazzling numbers and variety at our collecting lights; a feast for the eyes and CCD array of the scanner. Personally, I do not think we have the final answer to the beguiling question of why they end up stalled at lights.

**Figure 2:**
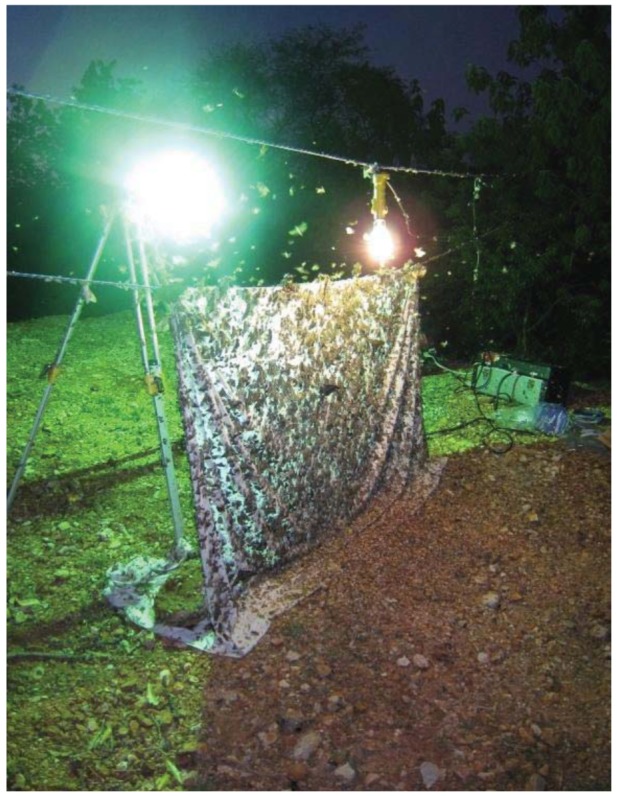
A good night mothing in Sonora. Moths of every description blanket the collecting sheet and their combined weight is starting to collapse the apparatus.

Moth collectors venture into remote areas with camping gear and automotive batteries or noisy and smelly gasoline-powered 110 V generators, mini power plants to energize one or more light bulbs. The choice of bulb type is usually a 400 or 800 Watt mercury vapor light bulb. Collectors should probably wear sunglasses or protective UV-glasses around these bulbs, they are that bright. They shine with not only all the visible wavelengths of light but a lot of light in the ultraviolet region of the spectrum which is strongly attractive to flying insects, especially moths. Some moth collectors also run short or long wave cylindrical black light fluorescent bulbs, but most now prefer the mercury vapor lamps. The lamp bulb, or two of them, are hung from a taut rope and placed one or two feet from the upper area of the white linen moth sheet. Thus, moths, beetles, flies, wasps and other insects can approach the sheet from opposite directions. The sheet is kept taught and at the bottom, about a foot or two of the cloth is formed into an “L” or foot and typically weighted down with medium-sized rocks. Moths and other insects fly toward the light, swirl around the bulb for a few minutes and typically land on the vertical face of the sheet, the foot, or somewhere in the near darkness within 10 or 20 feet of the lighted area. The pattern of moths resting on a sheet or nearby, and flying around the light is shown in Figure 2.

The moth sheets are usually operated all night with frequent topping off of the generator's gasoline tank. I recall a night of mothing early on with Joseph Scheer and Michael Wilson (from the Drylands Institute in Tucson) when I was tired and ready to call it a night around midnight. Joseph asked why I was retiring so early when the best moths had yet to fly. This amazed me. Although I'd been blacklighting, since high school excursions with collectors, mostly seeking beetles, with friends from southern California to the hot collecting spots of SE Arizona. It had never occurred to me that certain moths might fly very late at night or during early morning hours. It turns out that some of the most spectacular moths, including those colorful giants in the silk moth family Saturniidae fly very late, or early depending upon your perspective. All those years I'd gotten showy beetles but often missed out on the most flamboyant moths, the Saturniids. Who needs sleep?

**Figure 3:**
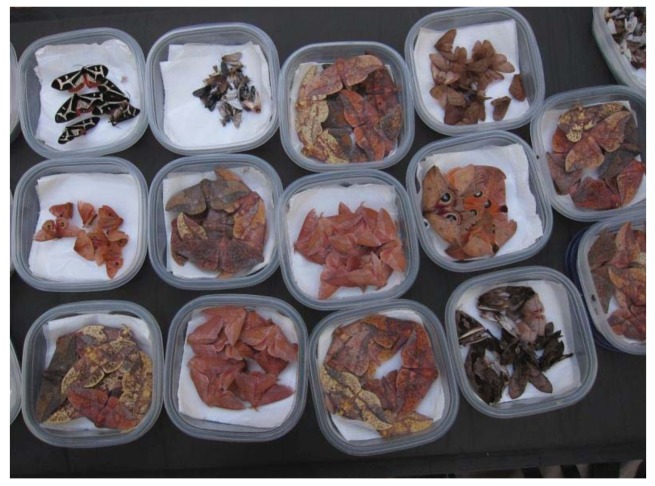
Freshly-collected moths in containers ready for spreading then scanning.

Moths are rarely collected with an insect net at the sheet. Joseph Scheer and other moth collectors usually have a number of wide-mouthed metal screw cap glass jars with Plaster of Paris bottoms at the ready. The plaster is charged at the start of each night with killing fluid, usually a big dose of ethyl acetate. This is the routine way to get the small moths. You spot a moth on the sheet you want, and approach it with the jar and quickly replace the lid. If you put the largest heavy-bodied moths like hawk moths (Sphingidae) or silk moths (Saturniidae) into a regular ethyl acetate killing jar, it will trash the other specimens, knocking off myriad wing scales on the smaller specimens. The larger moths are usually individually injected with ethanol and placed inside glassine envelopes or the plastic boxes to retain their pristine wing conditions for scanning. The night's catch is layered between soft tissue papers in plastic sandwich boxes (Figures 2 and 3) or similar containers and placed in a refrigerated cooler for the ride home. Joseph would often take stock of the night's catch laid out in open-topped boxes the next morning (Figure 3). If moths are not spread the next day, they are often frozen until they can be processed, days, weeks or months later. Freezing preserves their delicate colors and internal moisture. Some moth collectors also wear earplugs, not because the sound of thousands of moth wings is especially loud, but to keep the pesky creatures, or beetles or other flying insects, from mistakenly entering their ears and climbing toward their eardrums with horrifyingly loud sounds and discomfort. Joseph Scheer and Michael Wilson have both experienced this agony while mothing at the lights. Several entomologists have had to visit physicians to have their temporary ear canal resident evicted.

## 6. Specimen Preparation and Scanning Moths

Typically, butterflies and moths are “spread” right side up on a wooden spreading board. This is a centuries-old technique used by entomologists worldwide. In this procedure, you hold a fresh specimen in one hand, or forceps, and gently squeeze the thorax to spread open the wings a bit. With the other hand, you guide an insect pin into and through the thorax to the proper height. This is pinned into the midline slit of a balsa or similar soft wood spreading board. The fore and hind wings on one side are gently held back, and the forewing is moved by inserting an insect pin behind the strong forewing costal vein. This is pinned into the substrate while the hind wing is similarly moved and pinned. Then, artists vellum is placed over the wings and setting pins arranged just outside the wing edges. The spreading board with its moths is placed in a safe area at room temperature and allowed to dry for several days. When dry, the setting pins and vellum are removed and the moth specimen is labeled by adding a locality and ID label, the latter only if the species is known to the collector. The specimens are stored in dermestid-proof (scavenging beetles which will eat dried insects in collections) wooden boxes or glass-topped museum drawers in an insect-proof and moisture resistant insect cabinet in a home collection, or better, in an established insect museum with perennial care.

**Figure 4:**
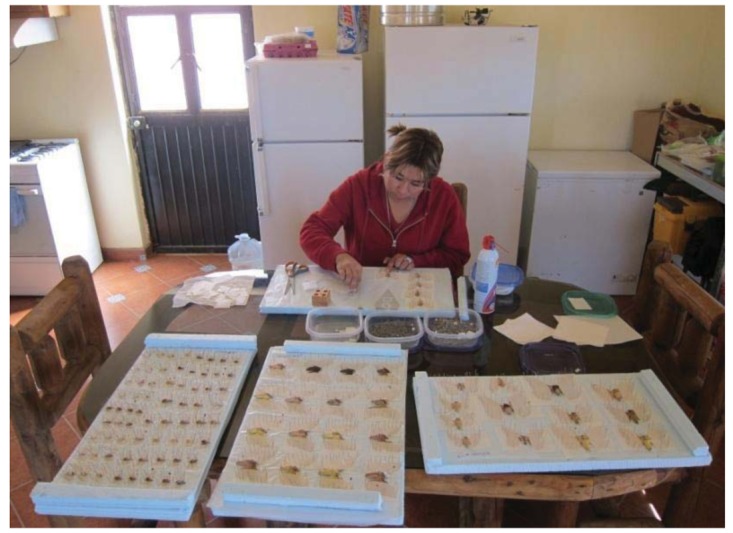
Scheer trained his housekeeper, Paz, one of the local Mayo indigenous people from near Las Bocas, to help with the mounting of the moth specimens.

Preparing moths for scanning is a very different procedure. Joseph Scheer developed his own customized methods. Typically, after a night's collecting, fresh and still pliable moths are removed individually from their plastic containers and pinned through the thorax but opposite from the spreading board method. Moths are pinned upside down upon high-density blue styrofoam boards. Their wings are moved with pins placed behind the strong anterior veins and aligned so that the hind margins of the forewings are more or less horizontal. The hind wings are brought forward and the antennae and front legs moved into position. The thin vellum sheets are pinned down over the wings and edged with more pins. Lastly, the thoracic setting pin is removed (Figure 4).

Once dry, the moths are carefully removed with lightweight broad forceps and placed on the scanner glass. Specimens are arranged in vertical rows across the scanner glass. They are held down with cleaned microscope slides. In the scanner software, Scheer selects a rectangular region around each moth, repeating the procedure and naming the files. The scanner(s) often run 24 hours a day as they scan first one specimen then the next. Originally, Scheer only focused and scanned the moths at one focal plane. Later, unsatisfied with having fuzzy abdominal edges or moth legs, he utilized the capability of the Creo scanners to scan at user-defined heights above the glass. Now, he typically makes 6 to 25 scans of the same moth but at different heights. At first, he painstakingly spent hours dragging “islands of the same focal plane” bits of the moth to another electronic canvas and built up the all-in-focus image. Today, he uses new capabilities in Adobe Photoshop or other multi-focal combining programs to accomplish this software sleight-of-hand maneuver. His prints now reveal magnified specimens but entirely in focus from top to bottom. Later, equally laborious corrections and color adjustments are made to each moth file in Photoshop prior to sending the CMYK file to the Iris for printing.

## 7. Mexico: The Fulbright Year

**Figure 5:**
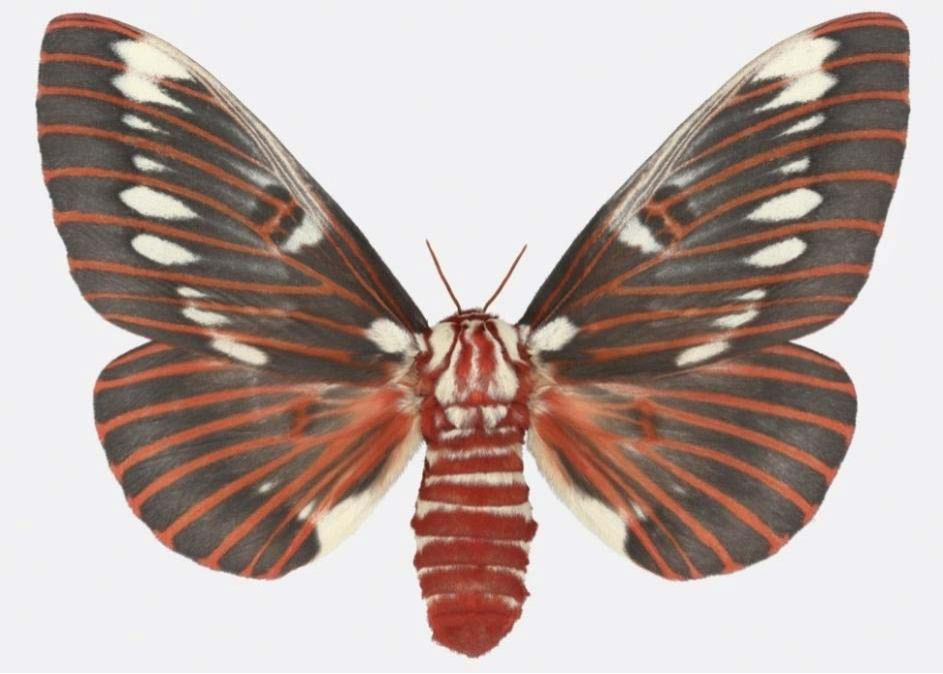
An Arizona beauty, the silk moth *Citheronia splendens*, a female.

**Figure 6:**
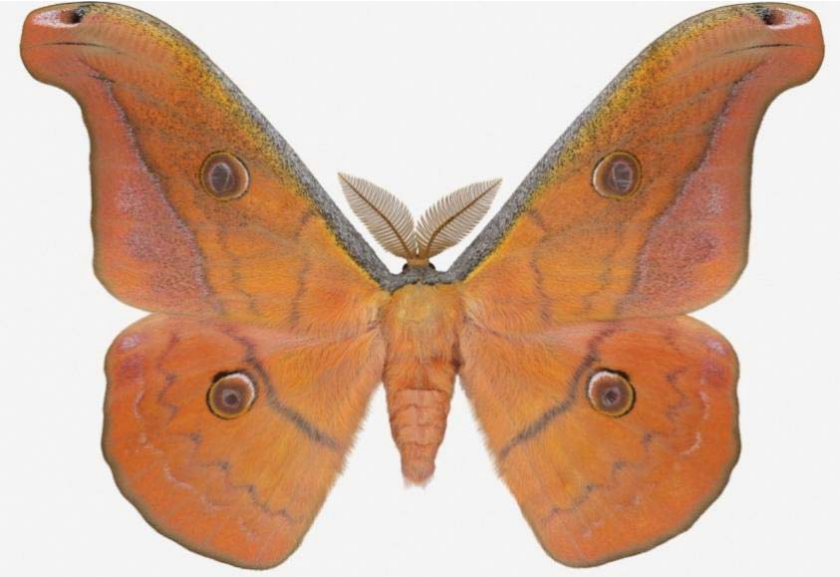
A large Sonoran silkmoth, *Copaxa lavandera* (Saturniidae).

**Figure 7:**
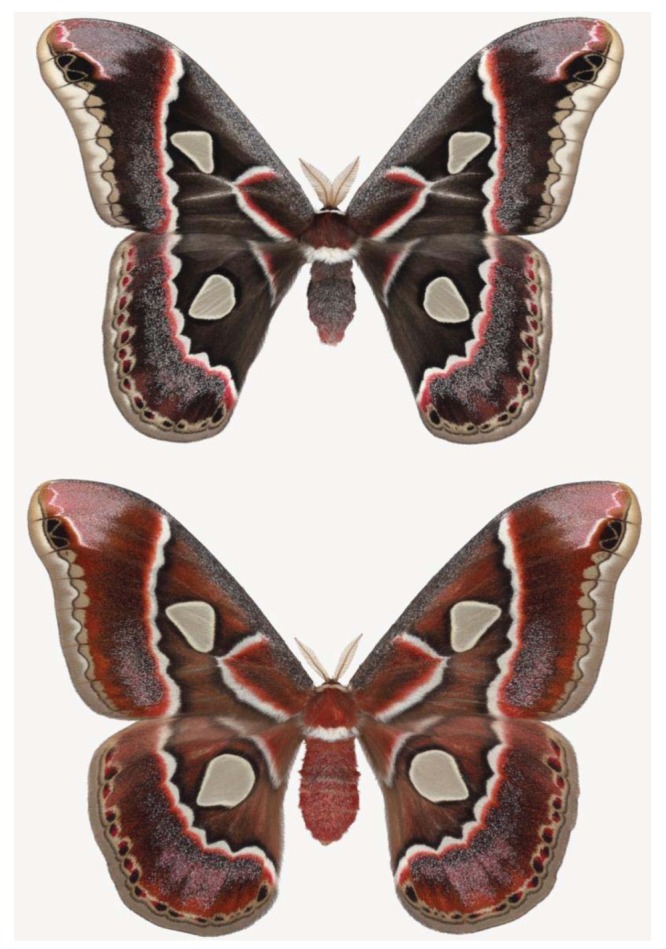
A male (upper) and female (lower) *Rothschildia cincta* silk moths.

**Figure 8:**
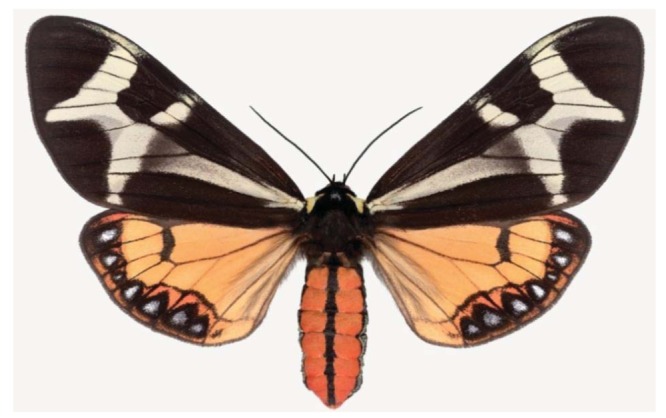
*Dysschema howardi*, an attractive tiger moth (Arctiidae).

**Figure 9:**
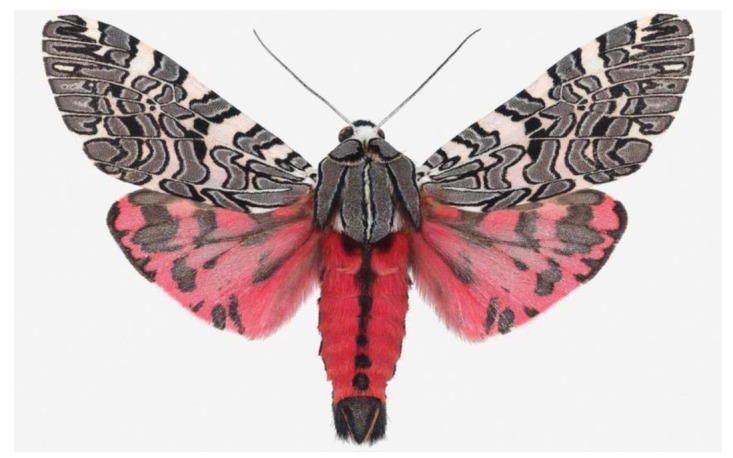
Another brightly colored Arizona tiger moth, *Arachnis picta*.

**Figure 10:**
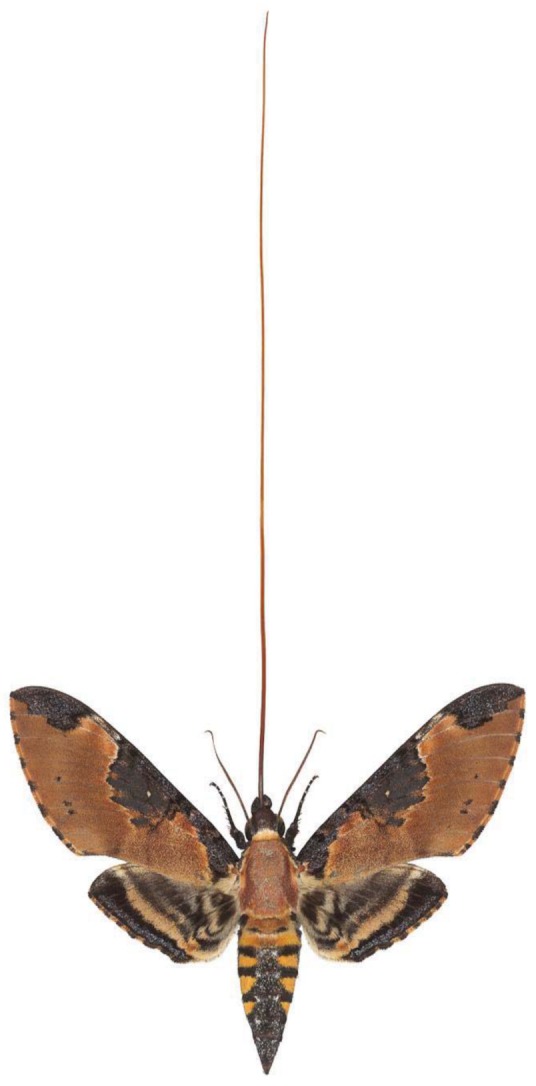
An extremely long-tongued hawk-moth, *Manduca ochus* from Sonora, Mexico.

For the past 5 years Joseph Scheer has made extensive visits to Arizona, making Tucson his base camp and organizing collecting expeditions around southern Arizona's canyons and into Northern Mexico, across the state of Sonora. These field trips were facilitated by local botanist and silk moth expert Michael Wilson, Director of the Tucson-based non-profit, the Drylands Institute [6] a decades old publisher of scholarly botanical books on the AZ/Mexico borderlands. As Drylands board president I've also been involved on many of these field trips and for logistical support while Scheer was in Tucson. These early mothing trips were exciting, and Scheer was amazed at the diversity of moths of all kinds, including the giant silkmoths, *Citheronia splendens* (Figure 5) and *Copaxa lavandera* (Figure 6), or *Rothschildia cincta* (Figure 7) of the Sonoran desert. Some of the other flamboyant moths collected during these expeditions included giant tiger moths *Dysschema howardi* (Figure 8) and equally striking *Arachnis picta* (Figure 9) or the long-tongued *Manduca ochus* (Figure 10). Many of the Sonoran moths were exhibited as large Iris prints by Scheer on a return trip to UNAM in Hermosillo, Sonora. An opening and evening talk on November 22, 2011 was followed by a temporary exhibit of his work during the all important study year.

Extensive trips were made into remote regions of Mexico and plans discussed for a dedicated full year of collecting during a sabbatical from teaching and advising duties at Alfred University. Perhaps, we hoped this dream would materialize with the funding and support of a Fulbright award. Eventually, the long wait from the Fulbright committee was over and Scheer had his grant.

By summer 2010 Scheer was moving collecting equipment and scanners to Tucson and storing them at my home. Trips into Mexico surveyed the best collecting areas and where to set up a makeshift research station. Eventually, the towns of Yecora (a mountainous region not far from Copper Canyon area) and a Mayo Indian beach town, La Bocas, near the larger city of Navajoa, Sonora, were selected as base camps. The Fulbright year ended in August, 2011 and Scheer made dozens of extended field trips into most of the diverse and often remote habitats in Sonora from sea level to 3,000 meters elevation in the northern mountains. This region as a whole is certainly the least biologically understood in North America. The scattered mountain ranges poke up from surrounding low deserts from SE Arizona into Sonora, and since Weldon Heald's book of the same name, the area has been termed the “Sky Islands.” Collectively, these moth collecting expeditions of Joseph Scheer and colleagues, the scanning, print-making and association with the Drylands Institute began to be known by us as the “Imaging Biodiversity” project. Two books are in preparation as a result of the extensive collections and scanned moth art. The first will be an extensive illustrated treatise on the 50 or so silkmoth species of AZ and Sonora, Mexico with detailed information on distribution, life history, food plants, seasonality and uses by indigenous peoples including the Mayo Indians of Sonora. Later, books will follow on the Sphingidae (hawkmoths) of the region, and other moth groups. As always, exciting new moth print exhibits are taking place in Mexico City, Colorado Springs and other gallery venues.

Once living in Mexico, Professor Scheer interacted with Mexican scientists [including his host, Francisco Molina-Freaner of Universidad Nacional Autónoma de Mexico's campus (UNAM) in Hermosillo, Sonora, Mexico] and importantly, lived among an indigenous culture, the Mayo Indians of coastal Sonora. Here were wonders indeed. Scheer was privileged to walk through forests of Pitaya and Hecho cacti with tribal elders, to eat, fish and cook with them. Who would guess that the epitome of death-defying Japanese cuisine (eating puffer fish, “Fugu”) was just another tasty morsel from the sea for the Mayo fishermen he befriended. Scheer was invited to attend special public and private ceremonies. He was able to photograph, video record and make audio tapes of the Fariceo, Vinado (deer) and Pascola dances between Easter and Lent. He asked about their beliefs and legends and explored their homelands and contemplates producing artful books on their ritualized dances and traditions. Unlike the Yaqui Indians whose deer dances are performed for the public in cities like Tucson, the Mayo perform theirs at individual homes and small communal spaces in the small villages and few ethnographers have chronicled their customs.

**Figure 11:**
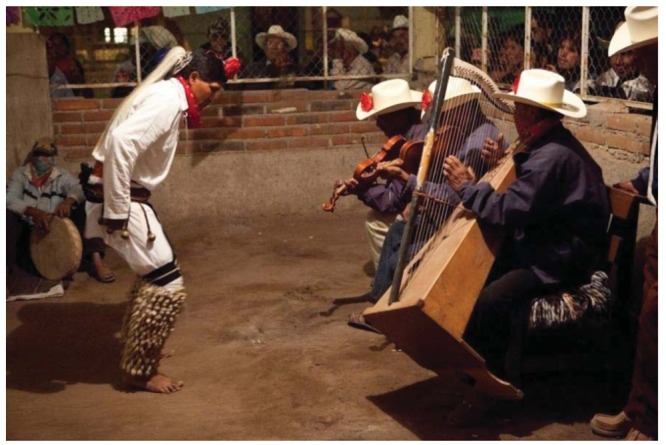
Mayo Pascola dancer, Masiaca, 2011. The dancer is wearing *Rothschildia* cocoons, known as ténaborim.

Scheer encountered a Mayo elder and healer living in Techive who was an expert at fashioning the deer dancer leg rattles from moth cocoons. It was his specialty, and everyone came to him for their strands. In the deer dances, the main dancer (wearing a deer headdress and shaking gourd rattles) also produces music from a belt made from spent brass ammunition cartridges or dried deer hooves. Other dancers including the Fariseos wear ankle rattles. The cocoon rattles are doubled over and tied to long strings. A really long string might have 400 cocoons and circle the ankle at the foot extending to just below the knees. The deer dancers stamp out mesmerizing beats almost like the buzz of a rattlesnake shaking out its warning (Figure 11). The cocoons are the tough silken pupal cases that protect silk moths during their long wait until emergence as adults on their quest to mate. The preferred cocoons gathered by the Mayo men in the wild are attached to dried plant stems. They belong to a gorgeous species of Sonoran silk moth, *Rothschildia cincta* (Figure 7). They are gathered by the hundreds and brought to the village rattle maker. The Mayo allows the male and female moths to emerge from their cocoons to fly back into the desert, assuring their conservation, and a continued supply of Mayo musical instruments.

**Figure 12:**
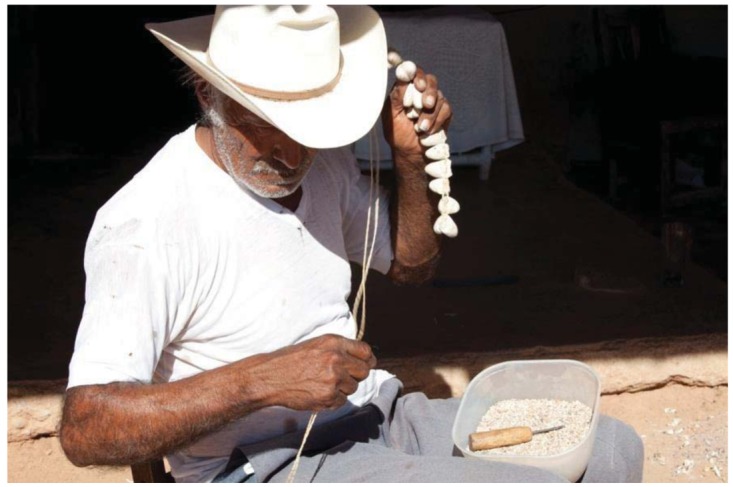
Chalo, a Mayo indian craftsman shakes and inspects a string of *Rothschildia* cocoons for just the right sound, effectively “tuning” the rattles.

The old man deftly selected which cocoons to use, favoring most but rejecting others (Figure 12). He did something else. Nearby was a small gourd bowl containing tiny white stones about the size of grains of wheat. He added five or more stones to each half cocoon, front and back before tying them to the main axis. He held them up to his ear and shook them, testing the sound from each. Often, he removed stones and replaced them with others, getting the “chhhit chhitt chhitt chhitt” sound just right. I learned from Joseph that these were no ordinary stones, and they had an odd entomological connection. Another insect order was involved. The rattle maker, Chalo, had gone outside the village and located nests of the painful stinging ants, seed harvesters or “pogos” (the genus *Pogonomyrmex*) and raided their tumuli, the trash and excavation dumps left by the ants. The ants seem to select similar-sized stones to fashion their crater nests. These made the rattle sounds inside the ténaborim. I was amazed to learn this other secret of the Mayo from Joseph. Michael and Joseph learned about other plants and animals from the Mayo including some of the food plants for their beloved silkmoths.

## 8. Exhibiting Moths on a Grand Scale

**Figure 13:**
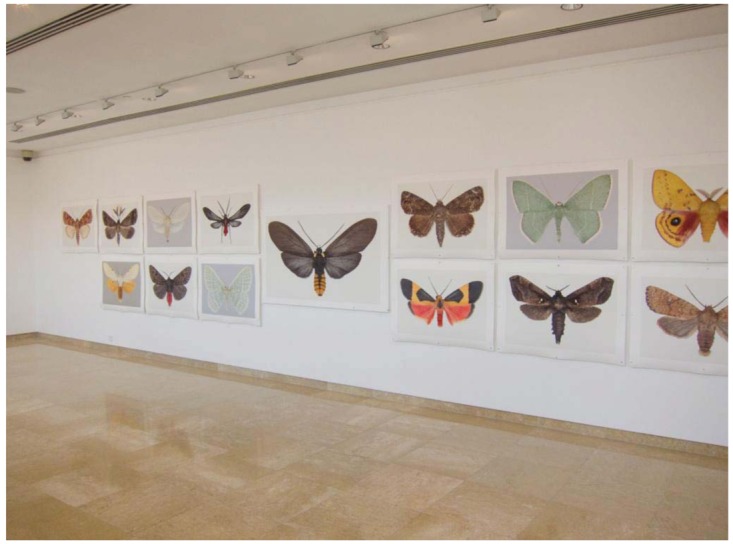
A display of prints in the He Xiangning Art Museum in Shenzhen, China.

Joseph Scheer has been exhibiting his Iris and Epson giant moth prints at Alfred University and in galleries and exhibits around the world for fourteen years. He's created massive 100-print one-man shows in Sweden and other large gallery exhibits throughout China (Figure 13), a favorite travel destination for locating the delicate handmade papers that he prints on, as well as many other countries besides galleries in New York City, Arizona, Colorado and others. He typically creates large (86 × 117 cm) Iris prints and offers them for sale as limited editions (signed and numbered sets of 10 each). Scheer never uses the same moth image again in a print series. Some of his work can be found online through keyword searches, including reviews and depictions of past exhibitions. If you find something you like, contact the artist directly at his Alfred University office.

Working with then Chief Curator Lisa Fischman of the University of Arizona Museum of Art (UAMA), Scheer was enticed in 2006 to visit Tucson for a solo show on campus. Fischman had worked with him at another museum in Atlanta during an earlier print exhibit. Over 40 giant prints were created including some of my favorites, the sphinx moths. Scans of adult *Manduca* (moths familiar to most gardens as the “horrid” green tomato hornworms) of several species were created about eight feet long from the tip of their long proboscides to the abdomen. They were phenomenal striking prints. Gallery viewers were in awe, standing next to basketball-player-tall hawk moths. Every detail and nuance of their morphologies, the patterns and textures of wing scales could be easily seen, accessible without a microscope or hand lens in the giant art prints. They became part of our world, we could now identify with them, and they were enlarged to human scale. Most of the prints during the UAMA Tucson show in 2006 were unframed, mounted underneath clear Plexiglass. Others were suspended vertically from ceilings.

Some of my favorites were stacks of elegant prints done on the surface of fragile diaphanous papers in stacks on a low table called “Moth book in three chapters.” From the sheaves of stacked prints were supporting wires leading up to a vertical display from the ceiling. Here were colorful Iris prints on handmade Yunnan paper. There was also electronic wizardry. A 20-channel ambient moth sound field, created with piezoelectric card speakers (along the wires at intervals) and a multichannel DVD source played moth sounds. Soft otherworldly moth talk confronted visitors who entered the gallery space. Other prints, known as sound prints, were moths on Goli paper with card speakers sewn into their lower hem and suspended vertically from low ceilings. The sound recordings were made from the actual sounds of the moth species depicted.

Most observers, even most scientists, were unaware that moths are capable of producing sounds. In reality, moth sounds are a combination of soft wingbeats along with rasping stridulation and even defensive hissing via air expelled forcefully through respiratory spiracles.

**Figure 14:**
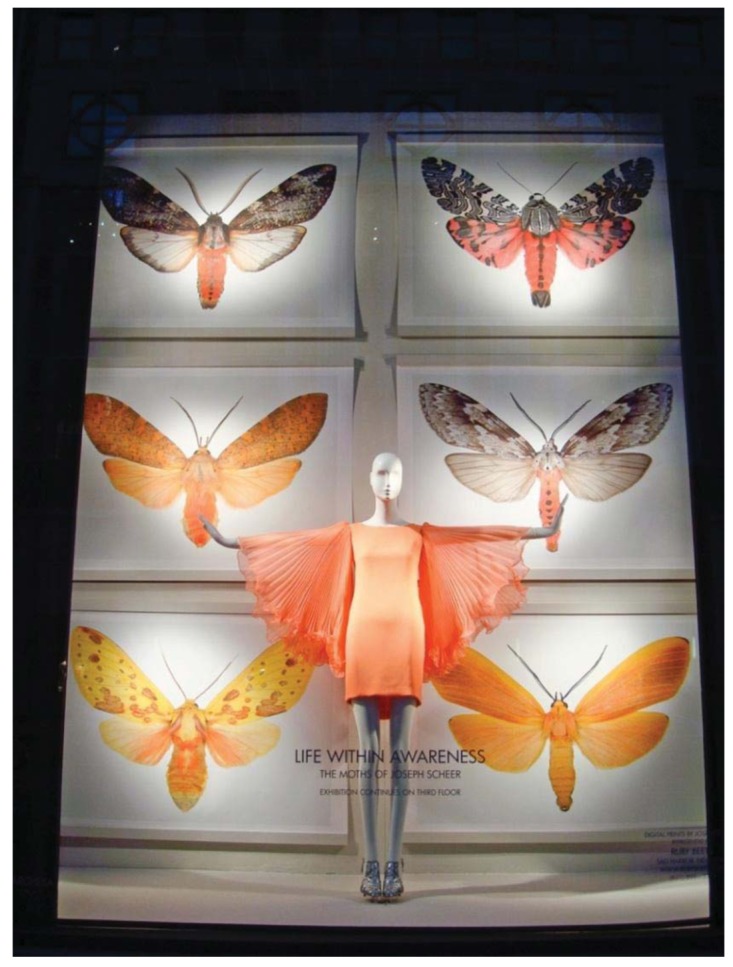
One of the Bergdorf-Goodman storefront windows on Fifth Avenue.

Haute Couture Moths? Some may have legs as slender as those of catwalk models, but a rare group of moths confronted passersby from the world-famous displays inside the five giant Bergdorf-Goodman windows facing Fifth Avenue in downtown New York City during 2010. It was a display by artist Joseph Scheer displaying his six-legged treasures (Figure 14). I imagine more than one person stopped to reflect and wonder what was going on. Is that a giant bug? What is it doing? I never realized they were so beautiful.

## 9. Conclusions

In western cultures, a moth is a small brown ugly thing that leaves holes in woolens stored in your closet, or so it seems. Moths are denizens of the dark nether world, after the sun sets, the playground of sprites and demons, at the very least a world of strange unsettling sounds, transient shapes and perhaps lurking danger. The death's-head moth of Europe (a group of three related *Acherontia* species) has a bold skull pattern emblazoned on its thorax. During the Middle Ages you certainly did not want one of these on your door, a very bad omen. Even worse, they could make a fearful hissing or squeaking sound when disturbed. Japanese film makers have their beloved giant “Mothra” battling other gargantuan creatures over fearful cities, and then there's that unsettling movie produced in the USA, “Silence of the Lambs.” The list goes on and on. Generally, the entertainment industry associate and provoke our deepest fears using moths as emblems or predictors of bad things. Butterflies are sweetness and light. Butterfly icons abound, on school lunch boxes, tee shirts, jewelry and bath soaps. Moths, seemingly, are more fitting for emblems of punk rock, alternative or other rock genres. They are the bad boys of the insect realm.

Moths are startling to behold. Looking at exquisite Scheer prints often evokes disbelief in first time viewers. Is that really a moth? Are they more colorful than many butterflies? Indeed they are. Scheer's prints are hyperrealistic, revealing details no camera or mere macro lens can capture. Many of the moth colors seem impossible, perhaps something leftover from the psychedelic 1960s, colors that were never supposed to go together. As expressed by Johanna Drucker: “Joseph Scheer's moths produce a sensation of wonder about the seemingly inexhaustible splendor of the world. We almost forget, looking at these prints, that these are not moths, but their simulacral phantom trace, created by techniques as complex as the subjects presented [4].” The images are not simply scientific illustration, although they share the tenet of super realism and exquisite details of master illustrators. They go far beyond; perhaps images indeed created or provoked by scientific pursuits yet answered by art.

One thing is for sure, the giant art prints of Joseph Scheer are changing the way people think about moths and insects in general. They are helping erase or modify unnatural fears and misconceptions about these fascinating creatures of the night, and day. Now, we see moths as non-threatening, even life-giving, in the case of the many long-tongued moth pollinators and their flowers.

## Figures and Tables

**Table 1:** A brief comparison of some high end graphical arts flatbed scanners.

**Brand/Model**	**Optical Resolution**	**D_max_^1^**	**Color Depth**	**Cost**
Creo/Kodak				
Eversmart Supreme	5600 dpi	4.3	48-bit color	$ 45,000
iQsmart3	3175 dpi	4.0		$ 20,000
Epson Expression				
10000 XL	2540 dpi	3.8	48-bit color	$ 2,500–$ 3,000

1 D_max_ is a measure of shadow detail obtained with the scanner's optics.
